# Flexible endoluminal robotic traction facilitates stable submucosal exposure during endoscopic submucosal dissection

**DOI:** 10.1055/a-2836-1430

**Published:** 2026-04-13

**Authors:** Kun Liu, Haoran Liu, Wendao You, Dongtao Shi, Deqing Zhang, Airong Wu, Rui Li

**Affiliations:** 1 74566 Department of Gastroenterology, First Affiliated Hospital of Soochow University, Suzhou, China

Endoscopic submucosal dissection (ESD) enables en bloc resection of superficial gastrointestinal lesions but remains technically challenging because of limited countertraction and unstable visualization of the submucosal plane, particularly during the early phase of dissection and in gravity-dependent locations 1 2 . We demonstrate the clinical application of a flexible endoluminal robotic traction system (EndoFaster), a detachable robotic traction system designed to overcome these challenges through joystick-controlled, multi-directional movement.

**Fig. 1:**
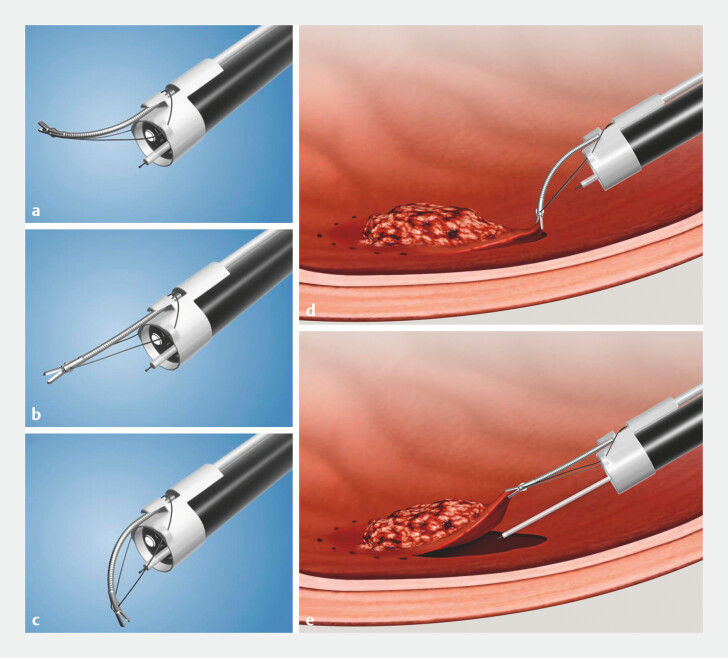
Working mechanism of the EndoFaster robotic traction system. **a–c** The EndoFaster robotic arm demonstrating multi-directional articulation, allowing controlled bending and positioning in different directions. **d, e** Schematic illustration of EndoFaster-assisted ESD. ESD, endoscopic submucosal dissection.

The system consists of a single-arm robotic module externally mounted on the distal shaft of a standard endoscope ( Fig. 1 ). The robotic arm runs parallel to the scope without interfering with the working channel. Two configurations are available for therapeutic gastroscopes and colonoscopes, differing in fixation sleeve diameter and robotic shaft length.

Unlike static traction methods, the robotic grasper allows for repeated tissue re-grasping, enabling the operator to dynamically adjust the traction vector as the dissection plane evolves. This ensures continuous, high-tension exposure of the submucosal space, facilitating a smooth transition from mucosal edge traction to deep inner-flap traction, regardless of the lesion's orientation or the effects of gravity.

**Fig. 2:**
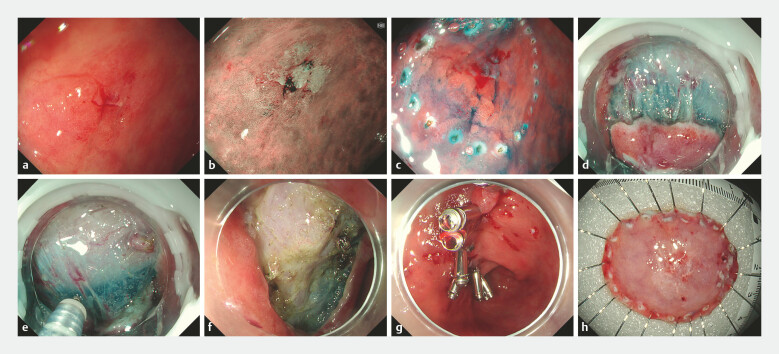
Representative EndoFaster-assisted ESD of a gastric antral lesion. **a** A flat lesion located at the lesser curvature of the gastric antrum observed under white-light endoscopy. **b** Magnifying narrow-band imaging revealing an irregular microvascular and microsurface pattern. **c** After lesion marking and submucosal injection. **d, e** EndoFaster-assisted traction providing stable elevation of the mucosal flap and clear exposure of the submucosal plane during dissection. **f** Post-ESD mucosal defect after en bloc resection. **g** The mucosal defect was closed using endoscopic clips. **h** The resected specimen retrieved and prepared for histopathological evaluation. ESD, endoscopic submucosal dissection.

**Fig. 3:**
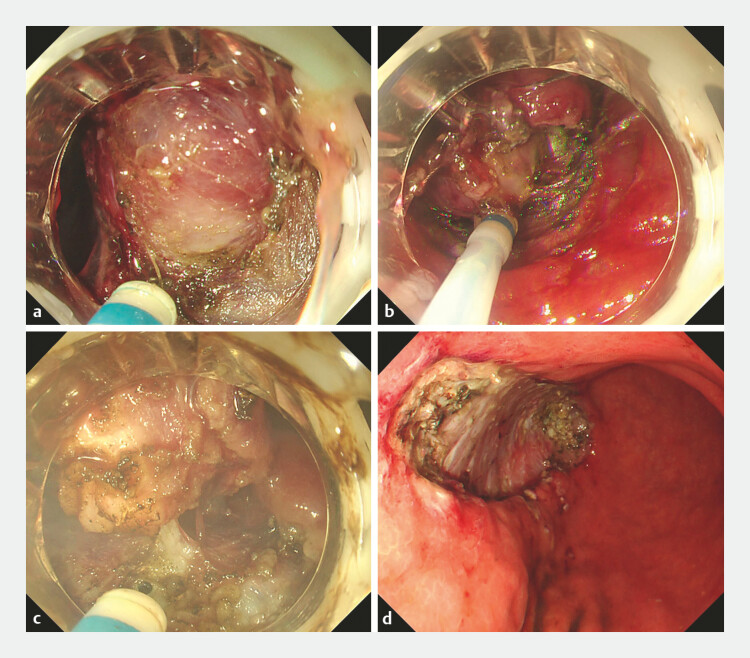
EndoFaster-assisted endoscopic resection of a gastric gastrointestinal stromal tumor (GIST). **a** Circumferential incision performed around the submucosal tumor. **b, c** Tumor dissection under EndoFaster-assisted traction. **d** Post-dissection wound bed showing a clean operative field without active bleeding.

The technique’s versatility was validated in eight consecutive procedures involving the esophagus ( *n* = 1), stomach ( *n* = 6), and colon ( *n* = 1; Table 1 and Fig. 2 ). In a representative gastric subepithelial tumor, the EndoFaster system enabled precise inward traction, clearly exposing the tumor–muscularis interface ( Fig. 3 ) and facilitating controlled dissection without perforation. Throughout this series, stable and adjustable countertraction was maintained across all anatomical sites, achieving a 100% R0 resection rate with no device-related adverse events ( Video 1 ).

**Table:** **Table 1** Patient characteristics and procedural outcomes of EndoFaster-assisted endoscopic submucosal dissection.

Case	Age/Sex	Location	Lesion size (cm2)	Histological diagnosis	Muscular injury	En bloc/R0 resection	Total procedure time (min)	Submucosal dissection time (min)	Adverse events
1	Mid 80s/M	Proximal gastric body (lesser curvature)	2.86	Low-grade intraepithelial neoplasia with intestinal metaplasia	No	En bloc, R0	21.3	11.2	None
2	Late 60s/M	High gastric body; cardia	6.00	Low-grade intraepithelial neoplasia with focal glandular atypia	No	En bloc, R0	38.2	18.8	None
3	Mid 50s/M	Transverse colon near hepatic flexure	1.00	Tubular adenoma	No	En bloc, R0	17.1	8.8	None
4	Late 50s/M	Gastric fundus	0.60	Leiomyoma	No	En bloc, margin indeterminate	8.5	4.2	None
5	Early 70s/M	Gastric angle/greater curvature	13.50; 1.80	High-grade intraepithelial neoplasia; GIST (low mitotic index)	Yes	En bloc, R0	46	28.9	Delayed bleeding (approximately one week after the procedure)
6	Mid 60s/F	Esophagus	13.50	Squamous carcinoma in situ	Mild	En bloc, R0	41.2	25.9	None
7	Early 70s/M	Gastric antrum	9.80	Differentiated-type adenocarcinoma, invasion to lamina propria	No	En bloc, R0	18.5	7.2	None
8	Mid 40s/F	Antrum–body junction (greater curvature)	23.40	Signet-ring cell carcinoma, intramucosal	No	En bloc, R0	32.1	20.6	None

EndoFaster-assisted traction facilitating submucosal exposure during ESD. ESD, endoscopic submucosal dissection.Video 1

While previous reports have demonstrated the feasibility of robotic traction in isolated cases 3 4 , our series highlights the versatility of the EndoFaster system across diverse gastrointestinal locations. Flexible endoluminal robotic traction provides stable and adjustable countertraction that may facilitate controlled submucosal dissection across various gastrointestinal locations and represents a promising technical adjunct for advanced endoscopic resection.

Endoscopy_UCTN_Code_TTT_1AO_2AG_3AD
